# Supplementary material for: Evaluating the reliability of functional near-infrared spectroscopy data in the context of a reasoning paradigm
Source: Dev Cogn Neurosci. 2026 Jun 18;80:101763. doi: 10.1016/j.dcn.2026.101763 (PMC13352402; doi:10.1016/j.dcn.2026.101763)
Supplement: Supplementary file 1 — Supplementary material [file mmc1.docx]

## **Supplementary Materials**

#### **S1. Participant Demographics**

| Measure | Item | Full Sample | Percentage (%) | Between Session Sample | Percentage (%) | Functional Connectivity Sample | Percentage (%) |
| --- | --- | --- | --- | --- | --- | --- | --- |
| Gender | Female | 62 | 73.8 | 17 | 65.4 | 24 | 66.7 |
|  | Male | 22 | 26.2 | 9 | 34.6 | 12 | 33.3 |
| Age | 18 | 2 | 2.4 | 2 | 7.6 | 5 | 13.9 |
|  | 19 | 8 | 9.5 | 3 | 11.5 | 3 | 8.3 |
|  | 20 | 21 | 25 | 3 | 11.5 | 10 | 28.8 |
|  | 21 | 22 | 26.2 | 6 | 23.1 | 7 | 19.4 |
|  | 22 | 12 | 14.3 | 2 | 7.6 | 1 | 2.7 |
|  | 23 | 11 | 13.1 | 3 | 11.5 | 2 | 5.6 |
|  | 24 | 2 | 2.4 | 2 | 7.6 | 2 | 5.6 |
|  | 25 | 1 | 1.2 | 0 | 0 | 2 | 5.6 |
|  | 26 | 1 | 1.2 | 0 | 0 | 0 | 0 |
|  | 27 | 2 | 2.4 | 2 | 7.6 | 3 | 8.3 |
|  | 29 | 2 | 2.4 | 3 | 11.5 | 1 | 2.7 |
| Education | High school | 43 | 58.9 | 16 | 61.5 | 28 | 77.8 |
|  | Bachelor's degree | 16 | 21.9 | 7 | 26.9 | 5 | 13.9 |
|  | Graduate degree | 7 | 9.6 | 1 | 3.8 | 0 | 0 |
|  | Some high school/GED | 7 | 9.6 | 2 | 7.8 | 3 | 8.3 |
| Race | White | 25 | 34.2 | 8 | 30.8 | 15 | 41.7 |
|  | Asian | 9 | 12.3 | 5 | 19.2 | 6 | 16.7 |
|  | Chinese | 7 | 9.6 | 3 | 11.5 | 4 | 11.1 |
|  | Some other race | 5 | 6.8 | 2 | 7.7 | 3 | 8.3 |
|  | Not applicable | 3 | 4.1 | 3 | 11.5 | 1 | 2.8 |
|  | No answer | 3 | 4.1 | 1 | 3.8 | 1 | 2.8 |
|  | Asian Indian | 2 | 2.7 | 0 | 0 | 0 | 0 |
|  | White, American Indian or Alaska Native | 2 | 2.7 | 0 | 0 | 1 | 2.8 |
|  | Other Asian | 2 | 2.7 | 1 | 3.8 | 0 | 0 |
|  | Vietnamese | 2 | 2.7 | 1 | 3.8 | 0 | 0 |
|  | Filipino | 2 | 2.7 | 0 | 0 | 0 | 0 |
|  | White, Some other race | 2 | 2.7 | 1 | 3.8 | 2 | 5.6 |
|  | Korean | 1 | 1.4 | 0 | 0 | 0 | 0 |
|  | White, Asian Indian | 1 | 1.4 | 0 | 0 | 1 | 2.8 |
|  | Chinese, Vietnamese, Other Asian | 1 | 1.4 | 0 | 0 | 0 | 0 |
|  | Japanese | 1 | 1.4 | 0 | 0 | 1 | 2.8 |
|  | Black or African American, American Indian or Alaska Native, Asian Indian | 1 | 1.4 | 0 | 0 | 0 | 0 |
|  | Black or African American | 1 | 1.4 | 1 | 3.8 | 0 | 0 |
|  | Chinese, Korean | 1 | 1.4 | 0 | 0 | 0 | 0 |
|  | American Indian or Alaska Native | 1 | 1.4 | 0 | 0 | 1 | 2.8 |
|  | Don’t know | 1 | 1.4 | 0 | 0 | 0 | 0 |

**Supplementary Table 1**. Demographic information for all samples

#### **S2. Channel Information**

| ROI | Estimated Brodmann Area | Channel | EEG Cap Location | # Participants With Usable Data |
| --- | --- | --- | --- | --- |
| Left DLPFC | BA 9/46 | S4_D3 | F3-F5 | 70 |
|  | BA 9/46 | S4_D2 | F3-AF3 | 70 |
|  | BA 9/46 | S4_D4 | F3-FC3 | 62 |
|  | BA 9/46 | S5_D3 | FC5-F5 | 65 |
|  | BA 9/46 | S5_D4 | FC5-F3 | 55 |
|  | BA 9/46 | S5_D16 | FC5-FC3 | 35 |
|  | BA 9/46 | S2_D3 | AF5-F5 | 40 |
| Left RLPFC | BA 10 | S1_D1 | Fp1-AF7 | 73 |
|  | BA 10 | S1_D2 | Fp1-AF5 | 72 |
|  | BA 10 | S2_D1 | AF5-AF7 | 68 |
|  | BA 10 | S2_D2 | AF5-F3 | 70 |
| Left VLPFC | BA 45/47 | S3_D1 | F7-AF7 | 60 |
|  | BA 45/47 | S3_D3 | F7-F5 | 63 |
| Right DLPFC | BA 9/46 | S7_D7 | AF6-F6 | 45 |
|  | BA 9/46 | S8_D5 | F4-AF4 | 70 |
|  | BA 9/46 | S8_D7 | F4-F6 | 73 |
|  | BA 9/46 | S10_D7 | FC6-F6 | 65 |
|  | BA 9/46 | S10_D17 | FC6-F4 | 35 |
| Right RLPFC | BA 10 | S6_D5 | Fp2-AF4 | 71 |
|  | BA 10 | S6_D6 | Fp2-AF8 | 72 |
|  | BA 10 | S7_D5 | AF6-AF4 | 68 |
|  | BA 10 | S7_D6 | AF6-AF8 | 68 |
| Right VLPFC | BA 45/47 | S9_D7 | F8-AF8 | 65 |
|  | BA 45/47 | S9_D6 | F8-F6 | 69 |
| Left Superior Parietal | BA 7 | S12_D8 | CP1-CP3 | 24 |
|  | BA 7 | S12_D10 | CP1-P1 | 23 |
|  | BA 7 | S13_D10 | P3-P1 | 55 |
|  | BA 7 | S13_D20 | P3-CP3 | 24 |
|  | BA 7 | S12_D19 | P3-P5 | 23 |
| Left Inferior Parietal | BA 39 | S11_D9 | P3-PO3 | 53 |
|  | BA 39 | S11_D8 | CP5-P5 | 53 |
|  | BA 39 | S13_D8 | CP5-P3 | 28 |
|  | BA 39 | S13_D9 | CP5-PO3 | 55 |
|  | BA 39 | S13_D11 | P5-PO3 | 55 |
|  | BA 39 | S11_D18 | PO3-P5 | 53 |
| Right Superior Parietal | BA 7 | S14_D12 | CP2-P2 | 30 |
|  | BA 7 | S14_D13 | CP2-CP4 | 19 |
|  | BA 7 | S14_D21 | P4-P2 | 19 |
|  | BA 7 | S16_D13 | P4-CP4 | 33 |
| Right Inferior Parietal | BA 39 | S15_D12 | P4-P6 | 51 |
|  | BA 39 | S15_D14 | P4-PO4 | 28 |
|  | BA 39 | S16_D12 | CP6-CP4 | 18 |
|  | BA 39 | S16_D14 | CP6-P6 | 36 |
|  | BA 39 | S16_D15 | CP6-PO4 | 35 |
|  | BA 39 | S15_D22 | PO4-P6 | 25 |
|  | BA 39 | S16_D23 | P6-PO4 | 16 |

**Supplementary Table 2.** Channels with corresponding EEG cap locations, approximate Broadman area locations, and number of good participants for each channel.

#### **S3. Channel Signal Quality**


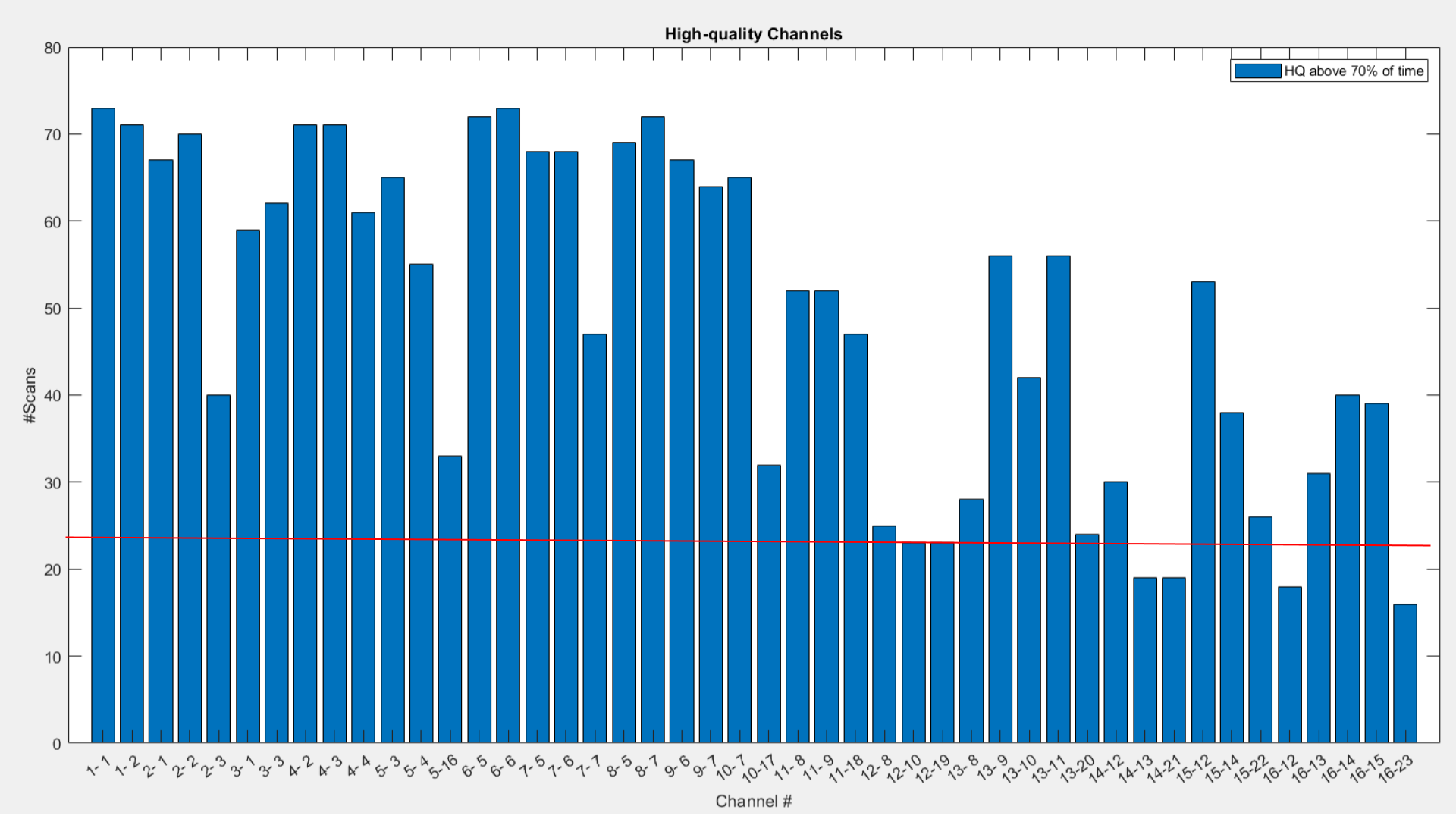


**Supplementary Figure 1.** Channel level plot of the number of participants with good quality data

per channel. The red line represents our exclusion criteria of a minimum of 23 good channels to be included

in the following analyses. The 4 channels that did not meet the minimum criteria were excluded.


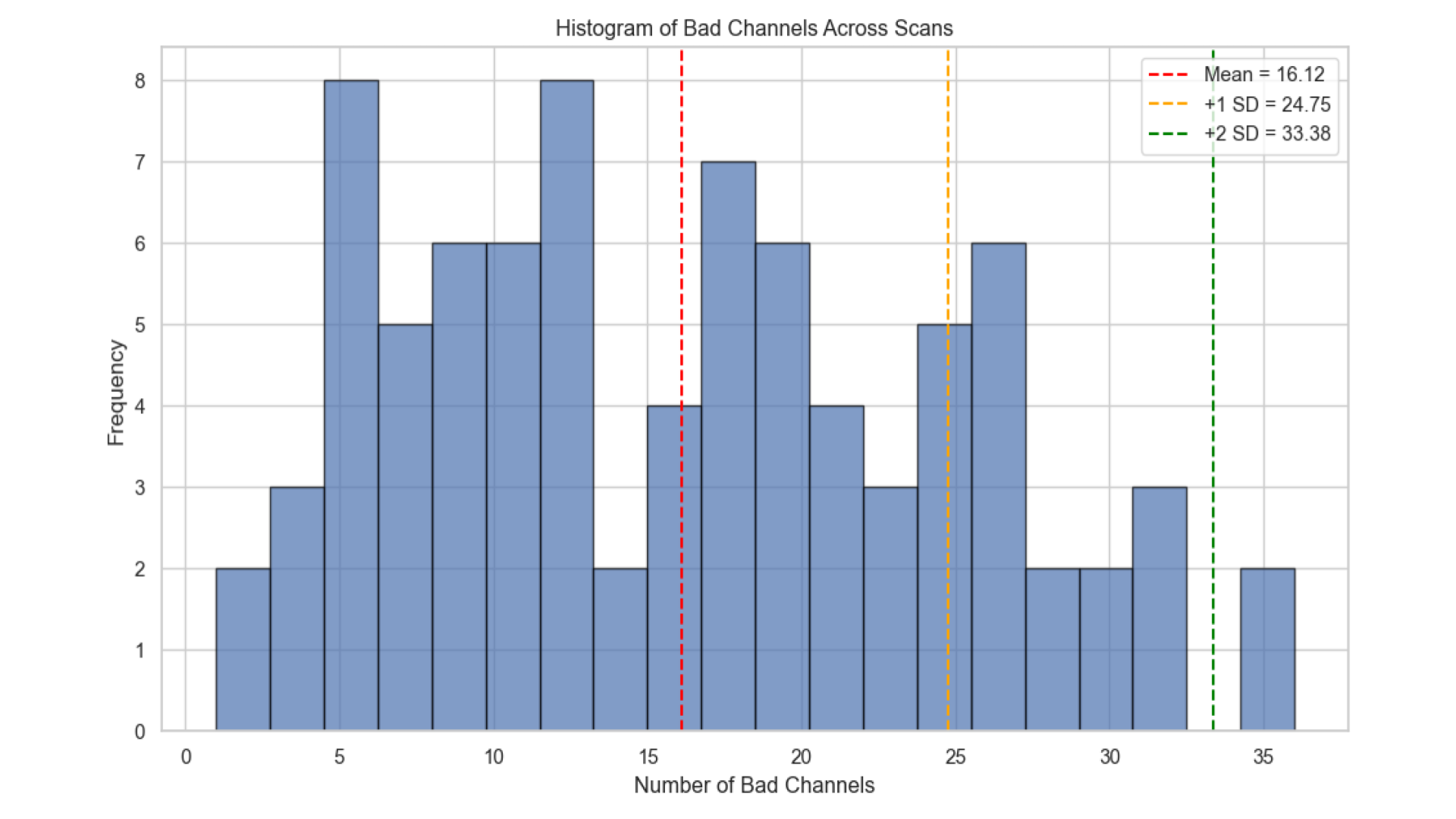
**Supplementary Figure 2**. Distribution of number of bad channels for full sample prior to exclusion (N = 84). Red dashed line is the mean of all bad channels across participants, gold line is 1 standard deviation above mean. Exclusion of participants based on the rounded value of 1 SD above the mean of bad channels (>27). If participants exceeded this threshold of 27 bad channels they were removed entirely from the analysis.

#### **S4. Behavioral Data**

###
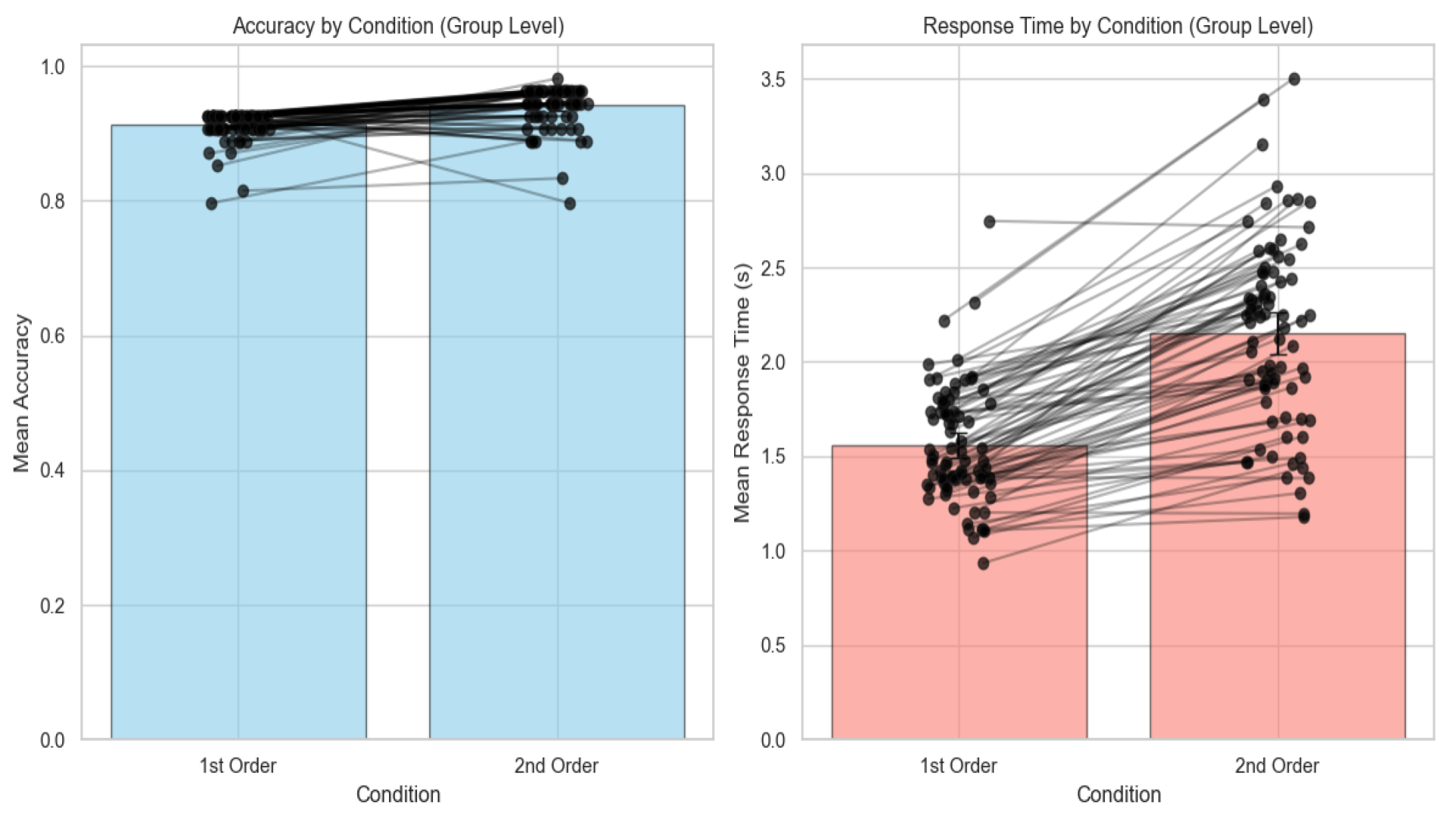


**Supplementary Figure 3.** Group level accuracy and response time by condition.

####

#### **S5. Task Activation for Individual Conditions**

***1st-order vs. Baseline contrast***

During 1st-order trials (Shape and Color conditions) relative to baseline, significant group-level activation (q < 0.05) was observed in 15 channels across bilateral frontal and parietal regions. These included channels positioned in such a way as to target left and right dorsolateral prefrontal cortex (DLPFC), left rostrolateral prefrontal cortex (RLPFC), bilateral inferior parietal cortex, and bilateral superior parietal cortex. The activation pattern indicates engagement of frontoparietal regions even during lower-order relational judgments.

***2nd-order vs. Baseline contrast***

For the 2nd-order (Match) condition, which required integration across multiple dimensions, we identified 14 significantly active channels (q < 0.05). Similar to Condition 1, activation was found bilaterally in DLPFC, RLPFC, VLPFC and Parietal Cortex, with additional engagement of more anterior prefrontal regions, compared to first order.


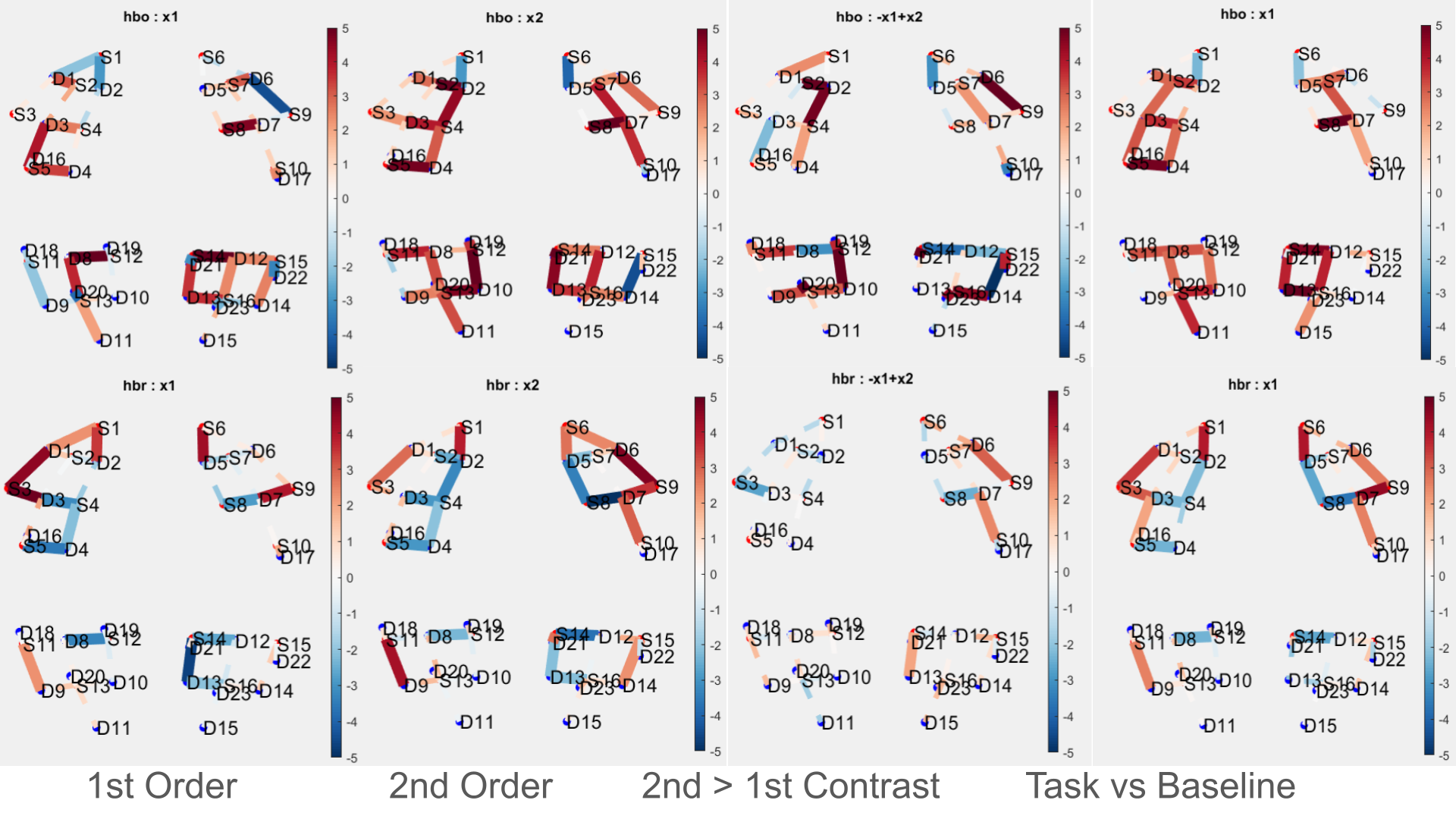


**Supplementary Figure 4.** Condition level HbO (top row) and HbR (bottom row) results. Indicates the relationship between HbO and HbR are not perfectly negatively correlated and there is some discrepancy between the two signals.

| Area | Channels | Task vs.Baseline  channel t-values | Task vs.Baseline  ROI t-values | 2nd vs.1st-order  channel t-values | 2nd vs.1st-order  ROI t-values |
| --- | --- | --- | --- | --- | --- |
|  | S1_D1 | t = 0.39, SE = 0.27, q < 0.794 |  | **t = 2.88, SE = 0.41, q < 0.03** |  |
| Left RLPFC (BA:10) | S1_D2 | **t = -2.29, SE = 0.24, q < 0.05** | **t = 2.93, SE = 0.33, q < 0.006** | t = .15, SE = 036, q < 0.90 | t = 1.79, SE = 0.55, q < 0.19 |
|  | S2_D1 | **t = 2.99, SE = 0.26, q < 0.016** |  | t = 1.76, SE = 0.39, q < 0.21 |  |
|  | S2_D2 | **t = 3.49, SE = 0.31, q < 0.005** |  | **t = 4.82, SE = 0.45, q < 0.000** |  |
|  | S6_D5 | t = -2.09, SE = 0.22, q < 0.08 |  | t = -2.27, SE = 0.34, q < 0.10 |  |
| Right RLPFC  (BA:10) | S6_D6 | t = 0.31, SE = 0.27, q < 0.827 | **t = 4.11, SE = 0.34, q < 0.000** | t = 1.,44 SE = 0.44, q < 0.32 | t = 1.65, SE = 0.55, q < 0.20 |
|  | S7_D5 | t = 2.22, SE = 0.32, q < 0.06 |  | t = .65, SE = 0.50, q < 0.65 |  |
|  | S7_D6 | **t = 2.92, SE = 0.30, q < 0.013** |  | t = 2.06, SE = 0.47, q < 0.13 |  |
|  | S4_D3 | **t = 3.63, SE = 0.19, q < 0.003** |  | t = 1.99, SE = 0.27, q < 0.15 |  |
|  | S4_D2 | t = 1.69, SE = 0.22, q < 0.16 |  | **t = 3.76, SE = 0.32, q < 0.003** |  |
| Left DLPFC  (BA:9/46) | S4_D4 | **t = 2.85, SE = 0.27, q < 0.014** | **t = 6.70, SE = 0.24, q < 0.000** | t = 1.19, SE = 0.40, q < 0.45 | t = 1.80, SE = 0.38, q < 0.19 |
|  | S5_D3 | **t = 3.15, SE = 0.24, q < 0.008** |  | t = -0.65, SE = 0.39, q < 0.65 |  |
|  | S5_D4 | **t = 4.83, SE = 0.24, q < 0.000** |  | t = .73, SE = 0.37, q < 0.62 |  |
|  | S2_D3 | **t = 2.99, SE = 0.16, q < 0.011** |  | t = 0.49, SE = 0.22, q < 0.72 |  |
|  | S7_D7 | **t = 3.23, SE = 0.16, q < 0.007** |  | t = 2.41, SE = 0.24, q < 0.08 |  |
| Right DLPFC  (BA:9/46) | S8_D5 | t = 0.68, SE = 0.26, q < 0.67 | **t = 6.66, SE = 0.23, q < 0.000** | t = 1.52, SE = 0.39, q < 0.32 | t = 2.13, SE = 0.36, q < 0.20 |
|  | S8_D7 | **t = 6.05, SE = 0.20, q < 0.000** |  | **t = 3.27, SE = 0.31, q < 0.01** |  |
|  | S10_D7 | t = 2.04, SE = 0.22, q < 0.08 |  | t = 1.43, SE = 0.35, q < 0.32 |  |
| Left VLPFC  (BA:45/47) | S3_D1 | t = 0.55, SE = 0.23, q < 0.72 | **t = 3.33, SE = 0.25, q < 0.002** | t = 1.49, SE = 0.38, q < 0.32 | t = 1.45, SE = 0.39, q < 0.235 |
|  | S3_D3 | t = 0.73, SE = 0.20, q < 0.65 |  | t = .75, SE = 0.32, q < 0.62 |  |
| Right VLPFC  (BA:45/47) | S9_D7 | t = -0.35, SE = 0.19, q < 0.81 | t = 0.67, SE = 0.25, q < 0.503 | t = 0.82, SE = 0.30, q < 0.59 | t = -0.18, SE = 0.40, q < 0.854 |
|  | S9_D6 | t = -1.12, SE = 0.25, q < 0.42 |  | **t = 3.29, SE = 0.40, q < 0.01** |  |
|  | S12_D8 | **t = 3.23, SE = 0.42, q < 0.007** |  | t = -1.46, SE = 0.60, q < 0.32 |  |
| Left SPL  (BA:7) | S12_D10 | **t = 2.73, SE = 0.41, q < 0.018** | **t = 5.60, SE = 0.43, q < 0.000** | **t = 5.37, SE = 0.52, q < 0.000** | t = 2.21, SE = 0.61, q < 0.176 |
|  | S13_D10 | **t = 3.77, SE = 0.30, q < 0.002** |  | **t = 3.02, SE = 0.44, q < 0.02** |  |
| Right SPL  (BA:7) | S14_D12 | **t = 4.71, SE = 0.41, q < 0.000** | **t = 7.34, SE = 0.43, q < .001** | t = -1.90, SE = 0.62, q < 0.18 | t = - .245 , SE = .62 , q < .853 |
|  | S16_D13 | **t = 5.70, SE = 0.38, q < 0.000** |  | **t = 2.62, SE = 0.56, q < 0.05** |  |
|  | S11_D9 | t = -0.40, SE = 0.32, q < 0.81 |  | t = .41, SE = 0.45, q < 0.77 | t = 1.40, SE = 0.48, q < 0.235 |
|  | S11_D8 | **t = 3.20, SE = 0.30, q < 0.007** |  | **t = 3.15, SE = 0.41, q < 0.02** |  |
| Left IPL  (BA:39) | S13_D8 | **t = 2.90, SE = 0.33, q < 0.013** | **t = 3.08, SE = 0.32, q < 0.004** | t = .19, SE = 0.44, q < 0.89 | t = 1.40, SE = 0.48, q < 0.235 |
|  | S13_D9 | t = 1.67, SE = 0.24, q < 0.16 |  | **t = 2.79, SE = 0.36, q < 0.03** |  |
|  | S13_D11 | **t = 3.72, SE = 0.21, q < 0.002** |  | t = 1.77, SE = 0.32, q < 0.21 |  |
|  | S15_D12 | t = 1.85, SE = 0.29, q < 0.12 |  | t = .24, SE = 0.38, q < 0.87 |  |
| Right IPL (BA:39) | S15_D14 | t = -0.18, SE = 0.35, q < 0.896 | **t = 2.50, SE = 0.34, q < 0.016** | **t = -2.88, SE = 0.50, q < 0.03** | t = -0.28, SE = 0.54, q < 0.854 |
|  | S16_D14 | t = 0.80, SE = 0.26, q < 0.602 |  | **t = 4.92, SE = 0.38, q < 0.000** |  |
|  | S16_D15 | **t = 2.30, SE = 0.23, q < 0.05** |  | t = 0.27, SE = 0.32, q < 0.85 |  |

**Supplementary Table 3**. Channel and ROI level task activation statistics for both the Task vs. Baseline and 2nd vs. 1st contrasts. Bolded values reached significance and survived multiple correction (q<.05)

#### **S6. Changes in Performance Across Cumulative and Binned Blocks**


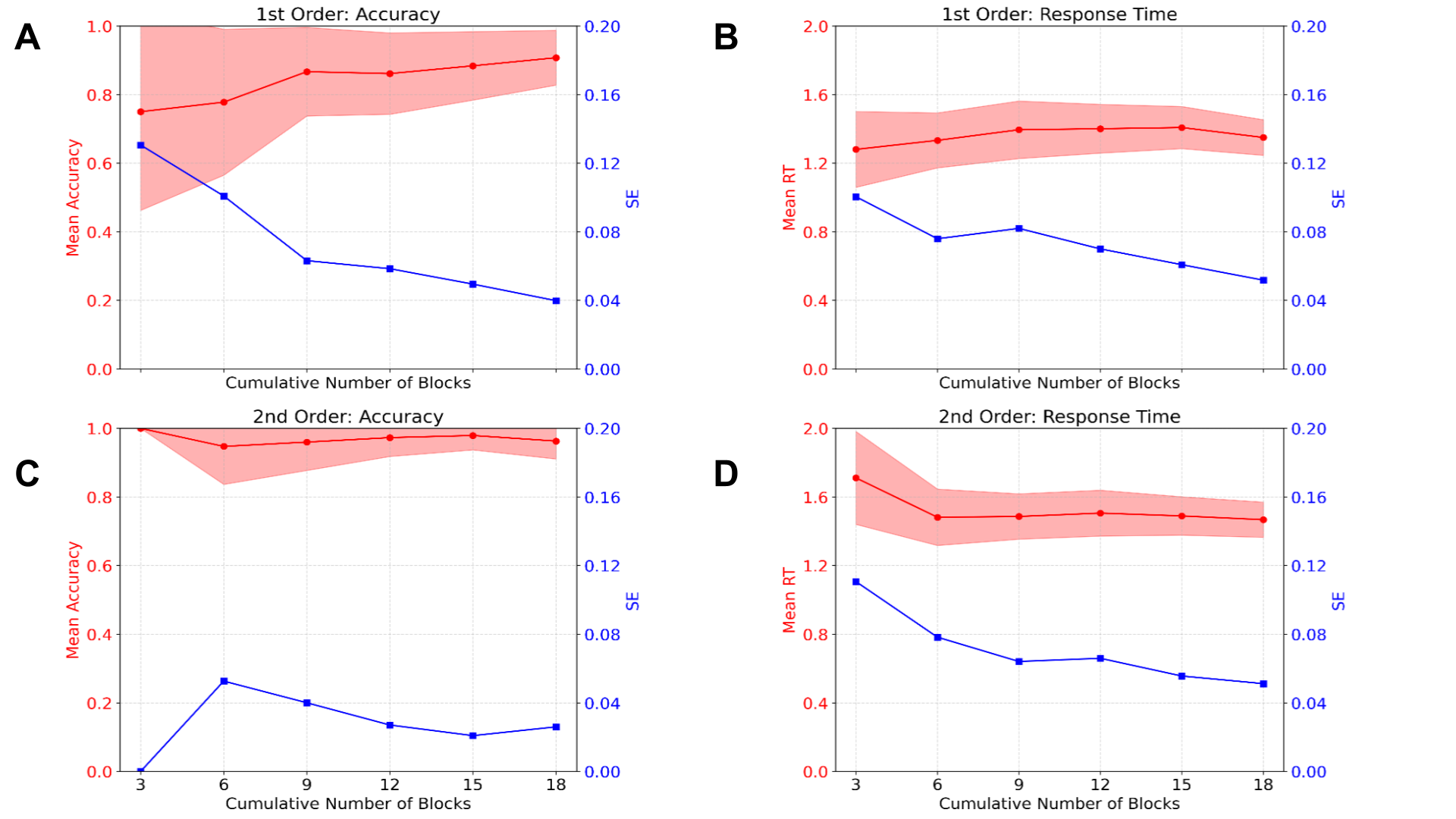


**Supplementary Figure 5**. Mean condition-level accuracy and response times as a function of cumulative number of blocks. Block order was randomized for each participant. There were no statistically significant changes in either condition for accuracy or response time across blocks. Solid lines represent the group mean across participants, and the shaded regions indicate ±1 standard error of the mean (SEM). The SEM was calculated across participants at each cumulative block level  **A**. 1st Order accuracy and SEM across cumulative blocks **B**. 1st Order response time and SEM across cumulative blocks 3. 2nd Order accuracy and SEM across cumulative blocks D. 2nd Order response time and SEM across cumulative blocks


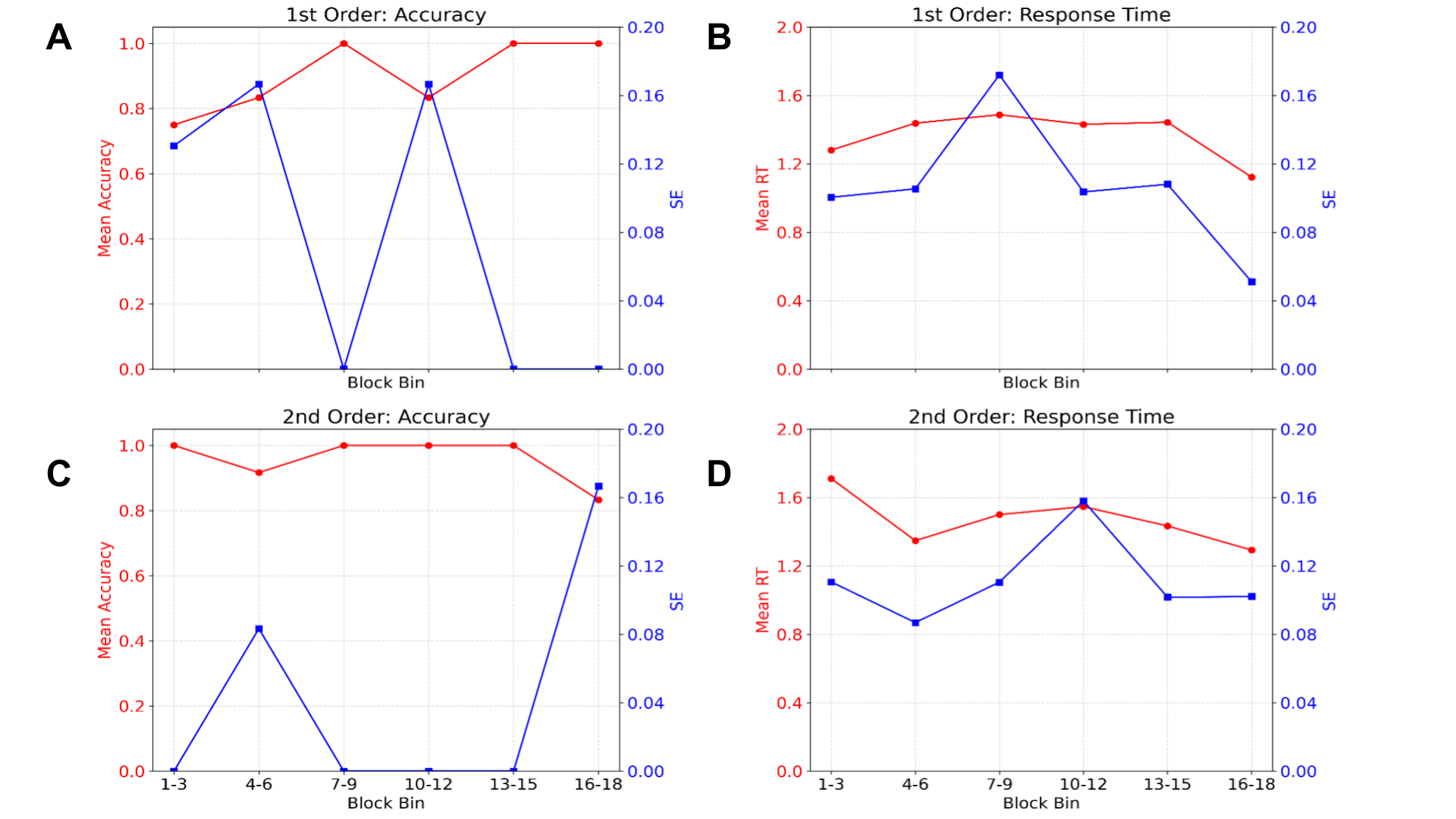


**Supplementary Figure 6**. Mean condition-level accuracy and response times as a function of binned blocks. Block order was randomized for each participant. There were no statistically significant changes in either condition for accuracy or response time across blocks. Red lines represent the group mean accuracy across the binned blocks, and the blue lines represent the group mean standard error across binned blocks. **A**. 1st Order accuracy and SE across binned blocks **B**. 1st Order accuracy and SE across binned blocks **C.** 2nd Order accuracy and SE across binned blocks D. 2nd Order accuracy and SE across binned blocks

#### **S7. Functional Connectivity at the ROI level**


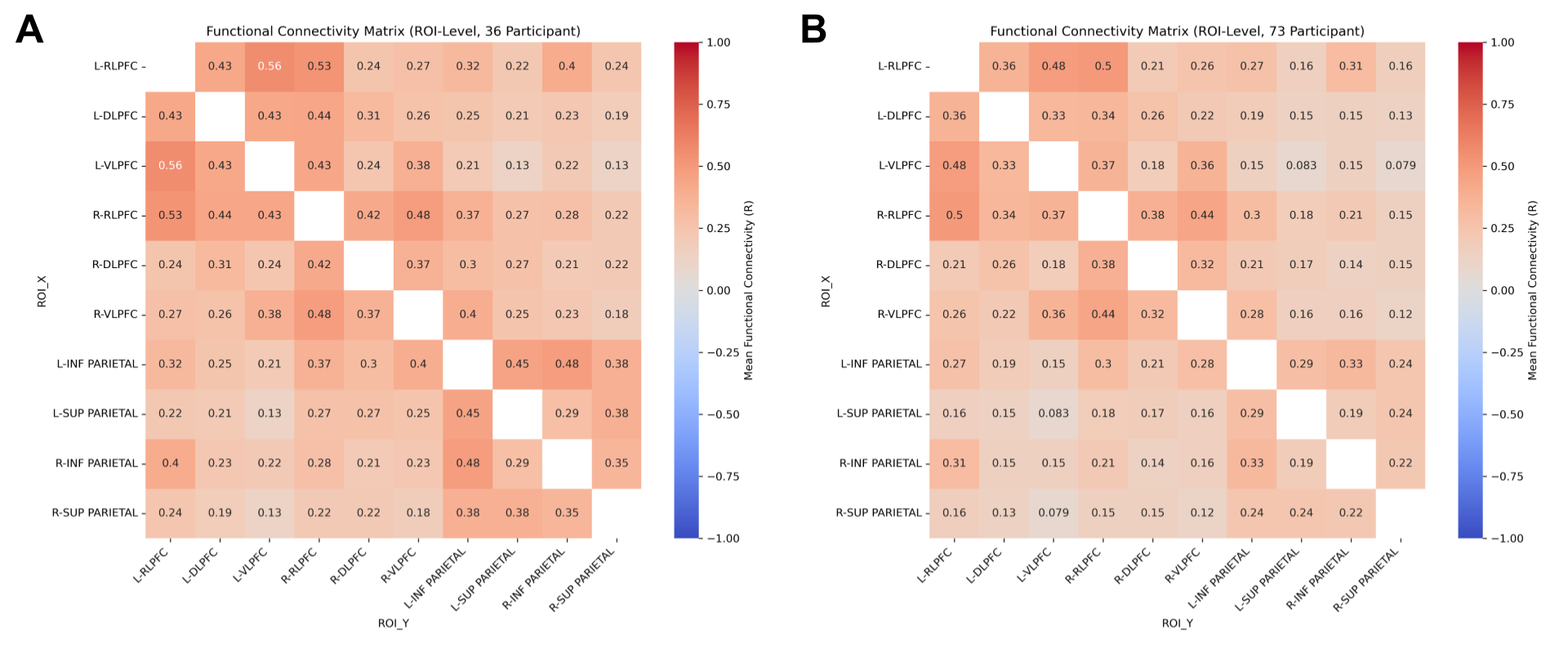


**Supplementary Figure 7.** Functional Connectivity correlation matrix at the ROI level. **A**.

Subsample of 36 participants. **B.** Full sample of 73 participants.

#### **S8. Channel Significance Status Across Blocks**


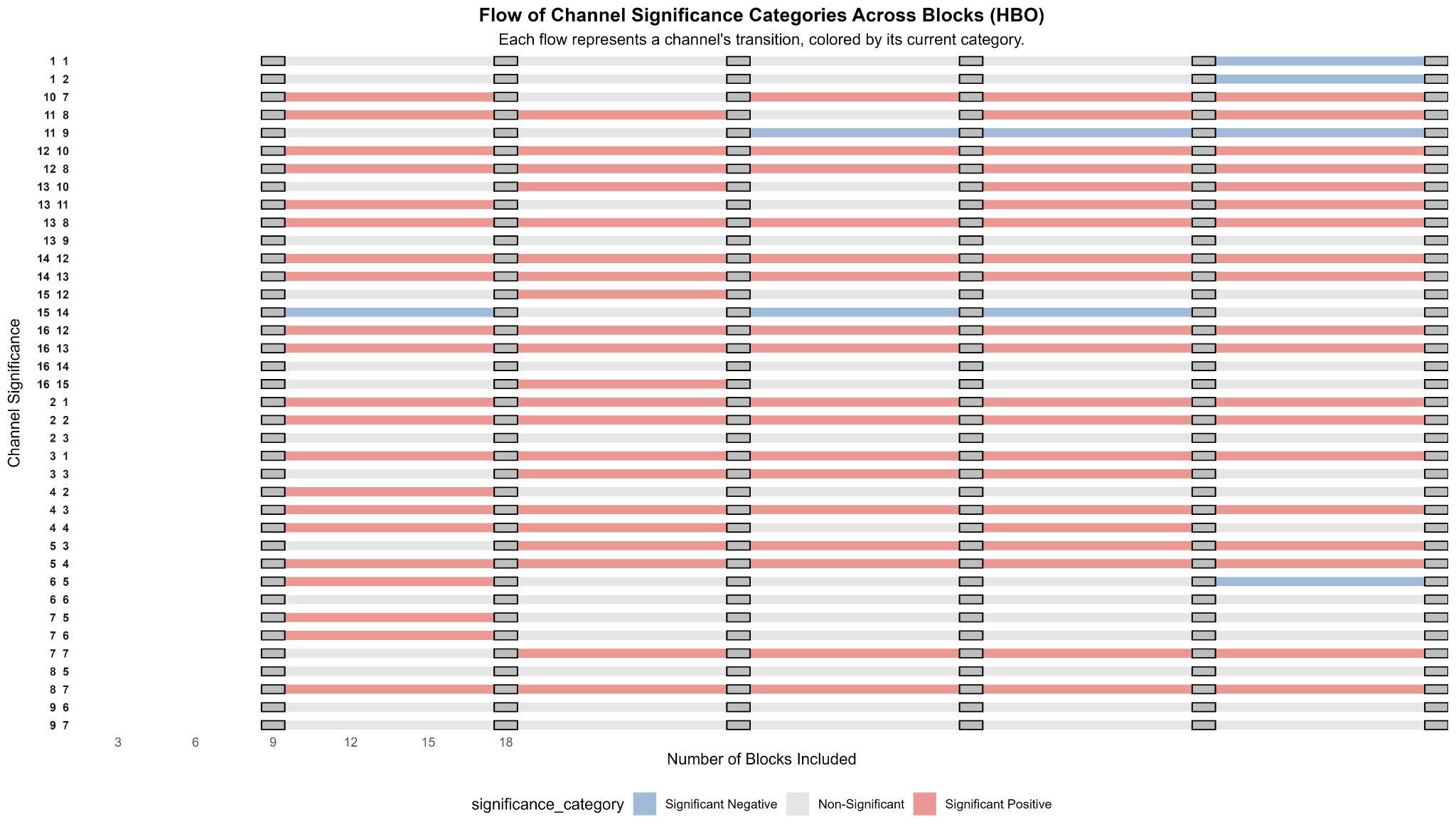


**Supplementary Figure 8**. Channel level data on the significance and sign of activation as a function of the

number of blocks included for the Task vs. Baseline condition.


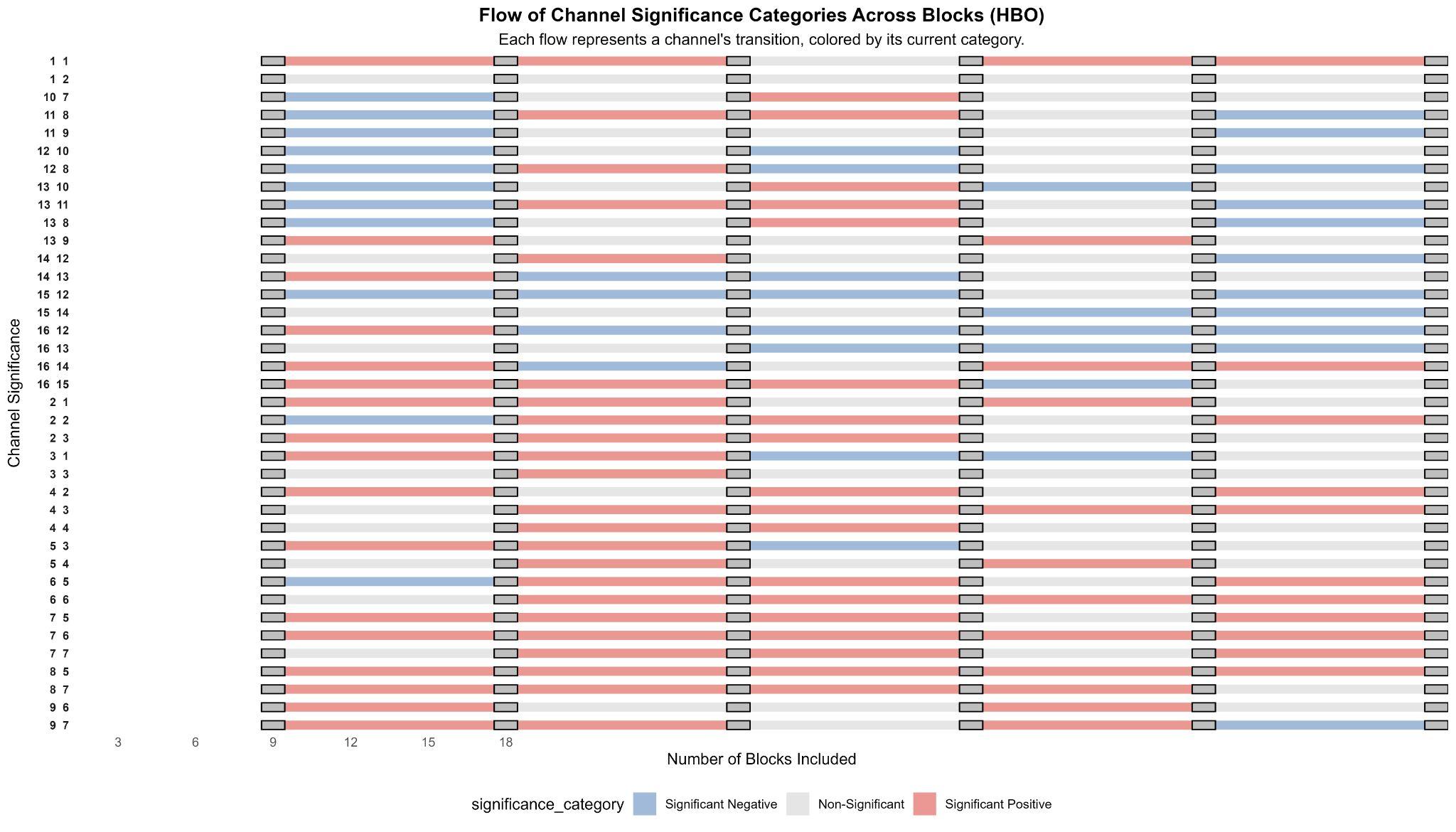


**Supplementary Figure 9**. Channel level data on the significance and sign of activation as a function of the

number of blocks included for the 2nd vs. 1st-order condition.

####

#### **S9. Comparison of Task Activation Across 1st and 2nd Halves of Session**

In addition to conducting split-half analyses across interleaved blocks to assess internal consistency reliability, we conducted parallel analyses comparing the 1st and 2nd halves of the dataset for all 73 participants who completed one testing session. In this way, we assess the degree to which activation changed over time, complementing the analyses examining the effect of cumulative block number.

***Group-Level Analyses***

At the channel level, Task vs.Baseline showed high stability across the 1st and 2nd halves (r = .65, ρ = .79, variance = .90, p < .001 across 36 channels), though this dropped substantially when limited to significant channels (r = .20, ρ = .33, variance = .27, p = .71). By contrast, the 2nd vs. 1st-order contrast showed low stability (r = –.25, ρ = –.66, variance = .35, p = .14), but with higher variance stability, indicating more consistency in signal magnitude than in activation patterns. Filtering to significant channels in this comparison also showed low stability (r = –.04, ρ = –.09, variance = .32, p = .90). At the ROI level, the Task vs. Baseline contrast again demonstrated strong internal consistency for the 1st vs. 2nd half comparison (r = .60, ρ = .74, *p* = .07, variance = .43). In contrast, the 2nd vs. 1st-order contrast yielded low and nonsignificant reliability for 1st vs. 2nd half blocks (r = –.46, ρ = .63, *p* = .22, variance = .47). Overall, both channel- and ROI- level stability for Task vs.Baseline was consistently high while the stability for both Task vs.Baseline and 2nd vs.1st-order contrasts remained low across spatial scales.

In sum, topographic visualizations of group-level beta maps revealed that the set of channels showing significant activation in the first half of the experiment often differed from those in the second half. While the task continued to elicit strong hemodynamic responses, the cortical regions most engaged changed over time, resulting in low split-half correlations. This pattern is likely to be explained by practice effects. As participants progressed through the session, their cognitive strategies may have shifted, enabling them to perform the task with greater automaticity or efficiency. Given that accuracy did not decline in the second session, this reorganization of the neural signal may reflect a change in strategy and/or neural adaptation across trials.


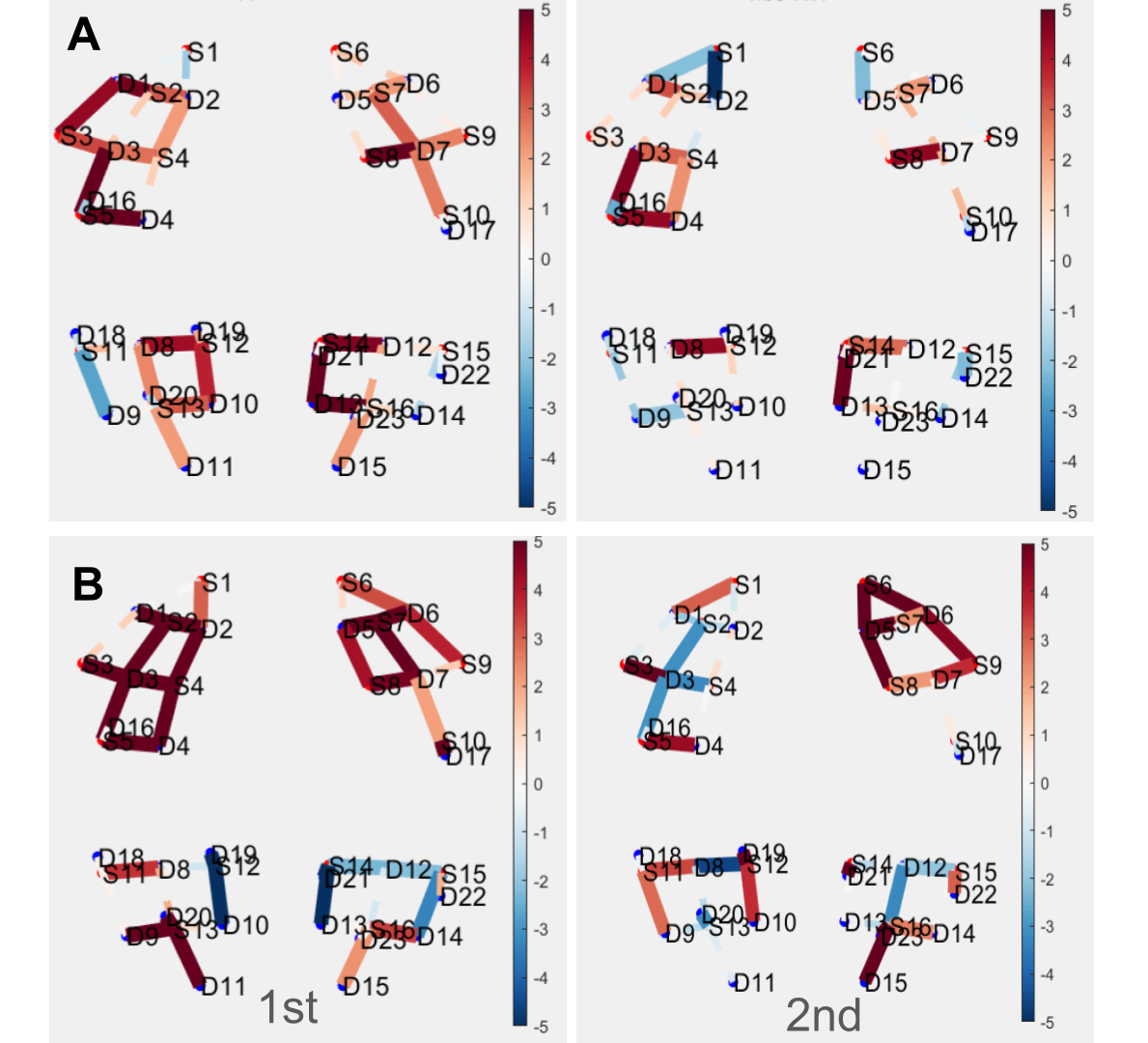


**Supplementary Figure 10.** Comparisons of 1st and 2nd half. **A**.1st half (left) 2nd half (right) Group level activation plots for the channel level Task vs.Baseline contrast. **B.** 1st half (left) 2nd half (right) Group level activation plots for the channel level 2nd vs.1st contrast.

***Participant-Level Analyses***

#### **S10. Within-Session Stability**

##### ***S10.1 Group Channel-level Task Activation Reliability for Interleaved Split-half Analyses***

At the channel level, the Task vs. Baseline group contrast showed high within-session spatial stability for interleaved blocks (r = .60, ρ = .75, variance = .97 p < .001). However, when filtering for significant channels, stability became non-significant (r = .49 ρ = .66, variance = .08, p = .40). For the 2nd vs. 1st-order contrast, interleaved blocks showed low spatial stability (r = .06, ρ =.12, variance = .44, p = .71); this was also true when including only channels with significant activation in both halves (r = .04, ρ = .09, variance = .63, p = .89). At the ROI level, Task vs.Baseline demonstrated near perfect internal consistency while the 2nd vs.1st-order yielded zero reliability. Overall, both channel- and ROI-level analyses revealed strong within-session spatial stability for Task vs. Baseline contrasts. However, the 2nd vs.1st-order relational complexity contrast showed no spatial stability, suggesting that contrast is likely not suitable for studying spatial distribution of activation, at either the channel nor ROI aggregation levels — or that a much larger amount of data would be needed to achieve stability.

##### ***S10.2 ROI-level Task Activation Reliability for Interleaved Split-half Analyses***

In addition to the channel-level results, we computed within-session reliability across ROIs, both at the group and participant levels. At the participant level, aggregating at the ROI level yielded similar results. The Task vs. Baseline contrast showed moderate internal consistency reliability (ICC = .56) with additional variance attributable to ROI (ICC = .09), but not the interaction between ROI and participant (ICC = .00). There was substantial variation in ICC values across channels (Supplemental Figure 12), ranging from ICC = .28 - .69. On average, individual ROIs in frontal locations (M_ICC_ = .61) had significantly higher ICC values than those in parietal locations (M_ICC_ = .40; t(8) = 3.27, *p* = .01). The 2nd vs. 1st-order contrast showed comparatively lower reliability (ICC = .47), with little-to-no variance attributable to channel (ICC = .00) or the interaction between channel and subject (ICC = .00). When calculating reliability per-ROI, there was a wide range (see Supplemental Figure 12; range = .00 - .56). On average, individual ROIs in frontal locations (M_ICC_ = .16) had significantly lower ICC values than ROIs in parietal (M_ICC_ = .38) locations (t(8) = -2.47, *p* = .04).

At the group level, the Task vs. Baseline contrast demonstrated strong internal consistency (r = .89, ρ = .94, *p* < .001, variance = .87). In contrast, the 2nd vs. 1st-order contrast yielded zero reliability (r = –.31, ρ = –.91, *p* = .38, variance = .18).

**
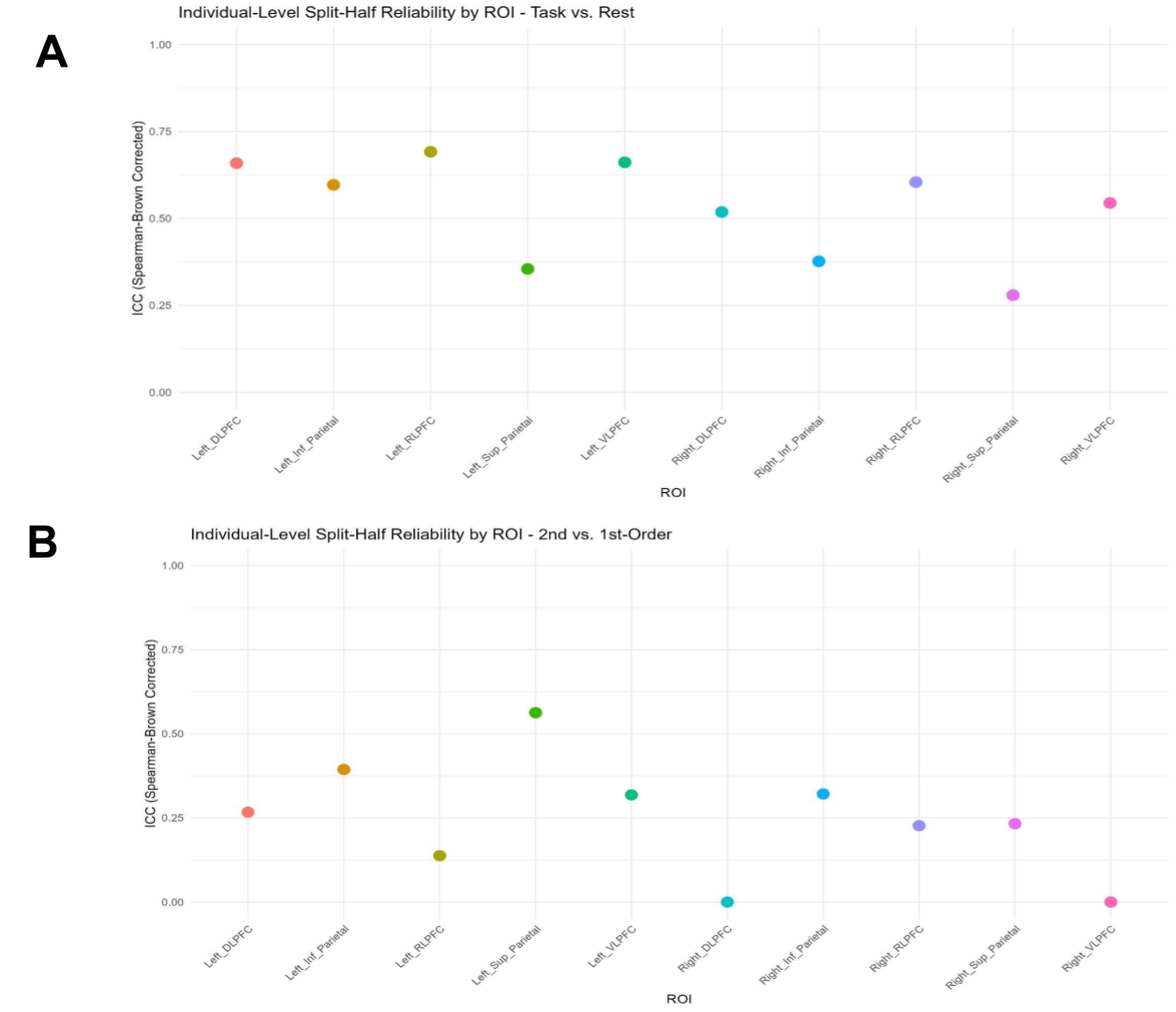
**

**Supplementary Figure 11.** Individual level ROI ICC’s for **A.** Task vs. Baseline **B**. 2nd vs.1st-order

##### ***S10.3 ROI-Level Functional Connectivity Reliability for Interleaved Split-half Analyses***

At the participant level, ROI-based correlations ranged from r = 0.95 to 0.99 (mean = 0.98, median = 0.99, SD = 0.01), again demonstrating strong reproducibility of individual connectivity profiles when signals are spatially aggregated. These results were consistent in the full 73-participant sample, where ROI-level correlations ranged from r = 0.96 to 0.99 (mean = 0.98, median = 0.99, SD = 0.01), with all participants exceeding r = 0.96. This pattern confirms the robustness of ROI-level connectivity as a stable and interpretable marker of within-session functional architecture.

At the group level for the subsample of 36 participants, ROI aggregation showed similarly high reliability (r = 0.998, p = 1.55 × 10⁻¹⁰⁴) to channel-level results, confirming that broader region-based estimates preserve within-session functional network topology. These findings were further corroborated in the full sample of 73 participants, where ROI-level connectivity correlations remained highly robust (r = 0.998, p = 7.03 × 10⁻¹⁰⁹), emphasizing the reproducibility of global network coordination within a single session.

#### **S11. Between-Session Reliability**

##### ***S11.1 Behavioral Data for Session 2***

Participants performed with high overall accuracy in both sessions. In Session 1, accuracy was 87.27% (SE = 0.0026) for 1st-order trials and 93.36% (SE = 0.0035) for 2nd-order trials. In Session 2, accuracy improved to 91.27% (SE = 0.0026) for 1st-order and 95.36% (SE = 0.0035) for 2nd-order trials. RTs consistently favored the 1st-order condition, with faster responses in both Session 1 (1.53 s vs. 2.02 s; SEs = 0.0345, 0.0559) and Session 2 (1.40 s vs. 1.71 s; SEs = 0.0345, 0.0559). Paired-sample t-tests revealed significantly faster RTs in Session 2 than Session 1 (t(n–1) = 5.16, p < .001), suggesting a practice or familiarity effect. Accuracy differences were marginal (t(n–1) = –2.02, p = .054), with a trend toward improvement in Session 2.

##### ***S11.2 Task Activation in Sessions 1 and 2***


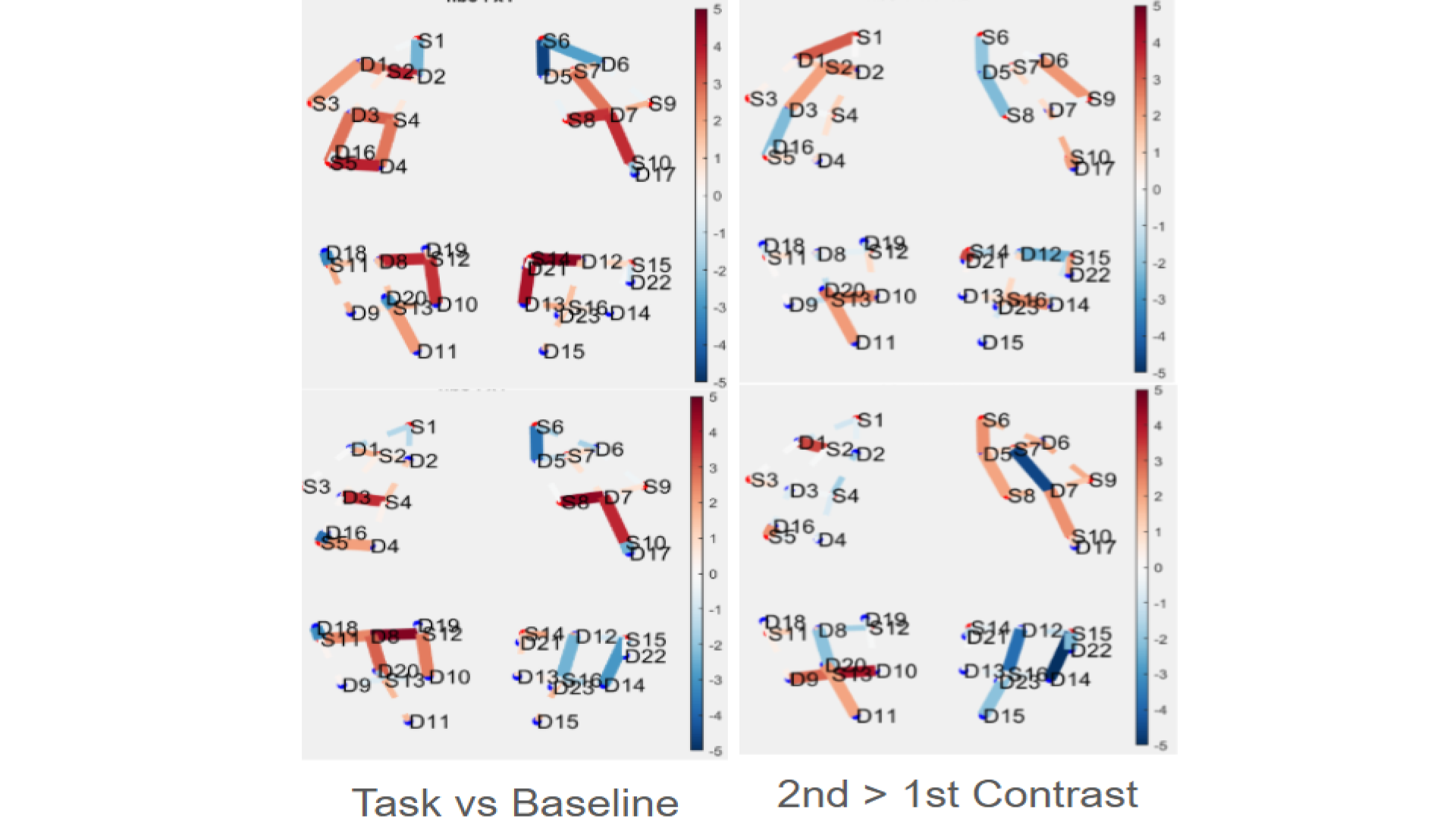


**Supplementary Figure 12.** Channel x Channel group level activation plots for session 1 (top row) and session 2 (bottom row) for the task vs. baseline and 2nd vs. 1st-order contrasts.

##### ***S11.2.1 Task Activation Reliability at the ROI Level***

At the participant level, aggregating into ROIs yielded similar results to the channel-level results. The Task vs. Baseline contrast showed relatively low test-retest reliability (ICC = .24) with little variance attributable to ROI (ICC = .01). Condition-specific contrasts showed a different pattern of test-retest reliability: 1st-order activation (ICC = .20), with no variance attributable to channel (ICC = .00), whereas 2nd-order activation (ICC = .09), with little variance attributable to channel (ICC = .03), showed noticeably lower test-retest reliability. The 2nd vs. 1st-order contrast showed similar test-retest reliability (ICC = .23) with little variance attributable to channel (ICC = .02).

At the group level, spatial consistency of activation for ROIs was higher than at the channel level for Task vs. Baseline, but remained poor for the 2nd vs. 1st-order contrast. The Task vs. Baseline contrast showed strong test–retest reliability (r = .87, p < .001), with similarly robust correlations for 1st-order (r = .72, p < .002) and 2nd-order (r = .73, p < .002). The 2nd vs. 1st-order contrast remained unreliable (r = .28, p = .44). Variance correlations were strongest for Task vs. Baseline (r = .87, p < .001) and remained high for both 1st-order (r = .76, p < .002) and 2nd-order (r = .74, p < .002), while the contrast again showed poor reliability (r = .31, p = .381). The pattern of signal amplitude differed across sessions for the Task vs.Baseline contrast (t = 3.96, p = .004) and the 2nd vs.1st-order contrast (t = 4.90, p = .001) indicating higher ROI level activity in session 1 than session 2.


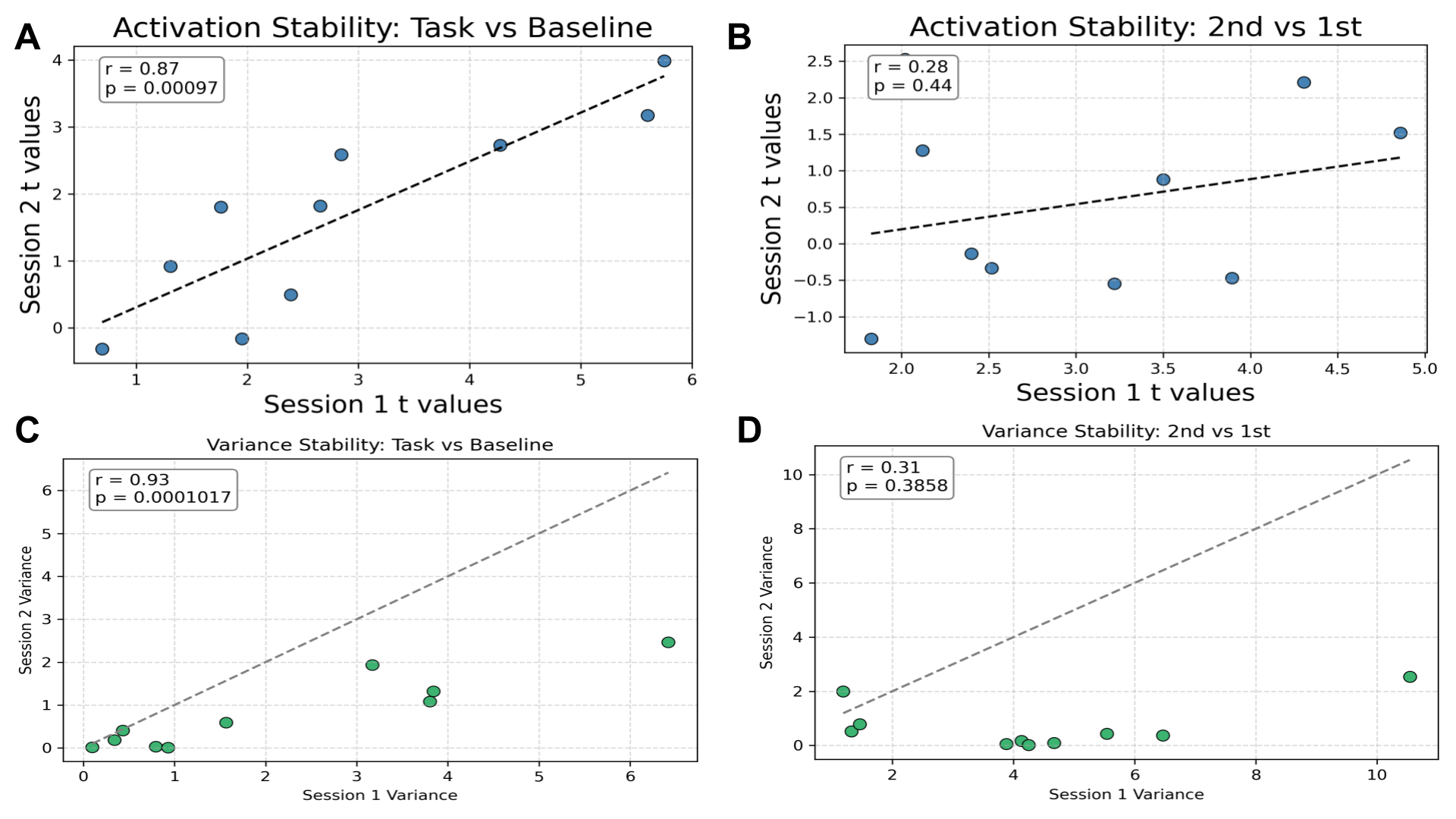


**Supplementary Figure 13.** Group-level between session activation and variance stability for ROI data. **A.** Task vs. Baseline activation stability **B.** 2nd vs. 1st-order activation stability. **C.** Task vs. Baseline variance stability **D**. ROI level 2nd vs. 1st-order variance stability

##### ***S11.3 Functional Connectivity***

To assess the test–retest reliability of group-level functional connectivity, we compared full connectivity matrices across sessions at both the channel and ROI levels on the entire between session subsample (N = 26). This analysis was also performed on the subset of participants from both sessions who had the highest quality data (<8 bad channels) and the results were almost identical. All p-values were derived using permutation testing, confirming the statistical significance of the observed stability.

***S11.3.1 Functional Connectivity Reliability by Channel at the Group Level***

At the channel level, functional connectivity patterns were highly stable across all 630 unique channel pairs. Group-level correlations between sessions were exceptionally strong for the 1st-order condition (r = 0.96, p < 1 × 10⁻⁶⁶), 2nd-order condition (r = 0.95, p < 1 × 10⁻⁶⁶), and Task vs. Baseline (r = 0.97, p < 1 × 10⁻⁶⁶). To ensure these findings were not influenced by global signal (average correlation between all channels), we removed each participant's unique global signal from each session and recalculated the group level functional connectivity for each session. The correlation for centered r values was also extremely strong (r = .99, p < 1 x 10^-63^), indicating this effect was not driven by global signal correlation across sessions. These findings indicate near-perfect reproducibility of global and condition-specific network structures **(Figure 13A).**

##### ***S11.3.2 Functional Connectivity Reliability at the ROI-Level***

For participant level data at the ROI spatial scale, functional connectivity demonstrated even greater stability for individual participants than at the channel level. For 1st-order, correlations ranged from r = 0.49 to 0.98 (.89 +/- .11); for 2nd-order, from r = 0.46 to 0.97 ( .90 +/- .10); and for Task vs. Baseline, from r = 0.49 to 0.98 (.90 +/- .11). Ninety-four percent of participants showed correlations above 0.80 in all conditions, with all p-values < 0.001 (permutation test).

For group level data at the ROI spatial scale, similarly high stability was observed relative to the channel-level results. Group-level correlations of ROI-to-ROI connectivity between sessions were r = 0.98 for both the 1st-order (p < 4.50 × 10⁻⁶⁶) and 2nd-order (p < 1.47 × 10⁻⁶⁶) conditions, as well as for Task vs. Baseline (r = 0.98, p < 1 × 10⁻⁶⁶), confirming highly reproducible patterns of large-scale network coordination across repeated measurements.


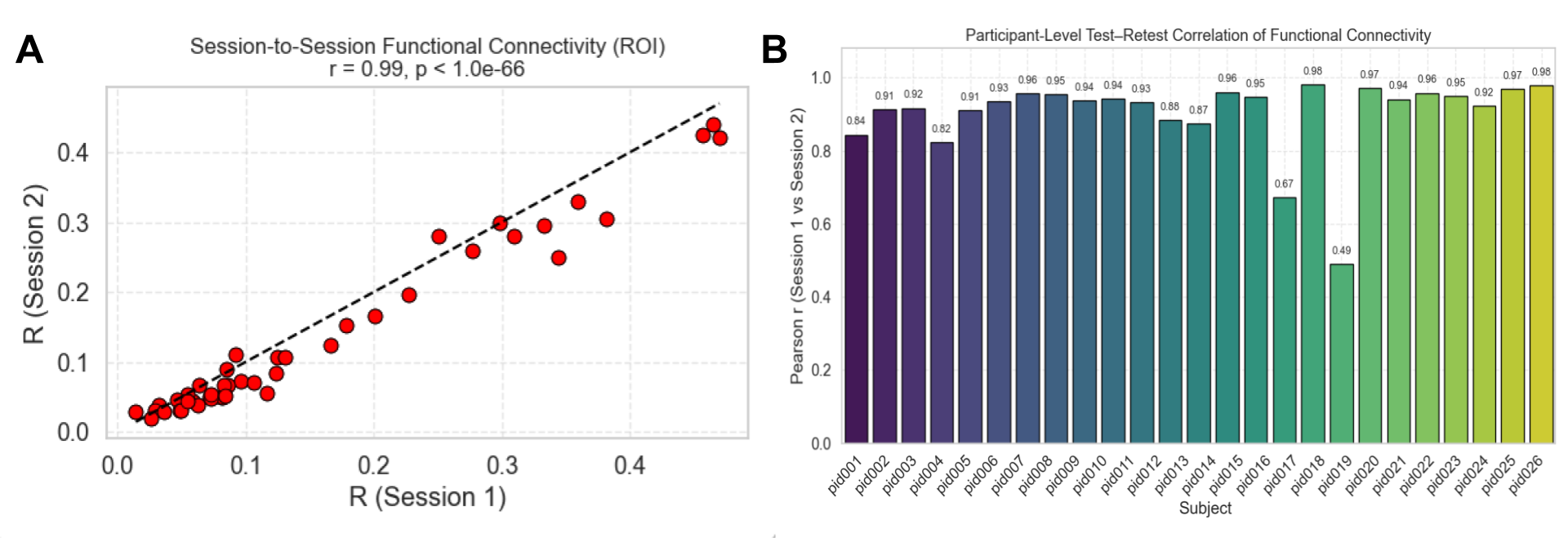


**Supplementary Figure 14. A.** ROI group level functional connectivity correlations from session 1 to

session 2 (r=.99) plotted against an identity line. **B.** ROI subject level functional connectivity correlations

from session 1 to session 2 with R values ranging from .49 - .98.

#### **S12. *Channel Quality as a Function of Hair and Skin Types***

An exploratory analysis was conducted to assess whether phenotypic characteristics—specifically skin tone, hair color, hair type, and hair texture—predicted fNIRS signal quality, as in prior studies (Holmes et al., 2024; Yücel et al., 2024). Here, we operationalized signal quality as the number of unusable (bad) channels identified during preprocessing. The results of this analysis should be considered with caution, as it was not fully powered to detect main effects or interactions; a larger and even more diverse sample would be needed to measure the combined effects of skin color, hair color, and hair texture. Participant-level metadata were compiled for 77 individuals, including manually coded phenotypic attributes and the number of bad channels calculated using the QT-NIRS module in the NIRS Toolbox. Skin tone was rated on a five-point ordinal scale based on visual inspection and video recordings, and recoded into four categories: Fair, Medium, Light Brown, and Brown. Hair color was categorized as Blonde, Brown, or Red/Black based on self-report and visual inspection. Hair type was classified by curl pattern (Straight, Wavy, Curly, Kinky), and hair texture was categorized as Fine, Medium, or Coarse. Participants with missing data for any variable were excluded.

A factorial linear model was constructed with the number of bad channels as the dependent variable and skin tone, hair color, hair type, and hair texture as fixed-effect predictors.


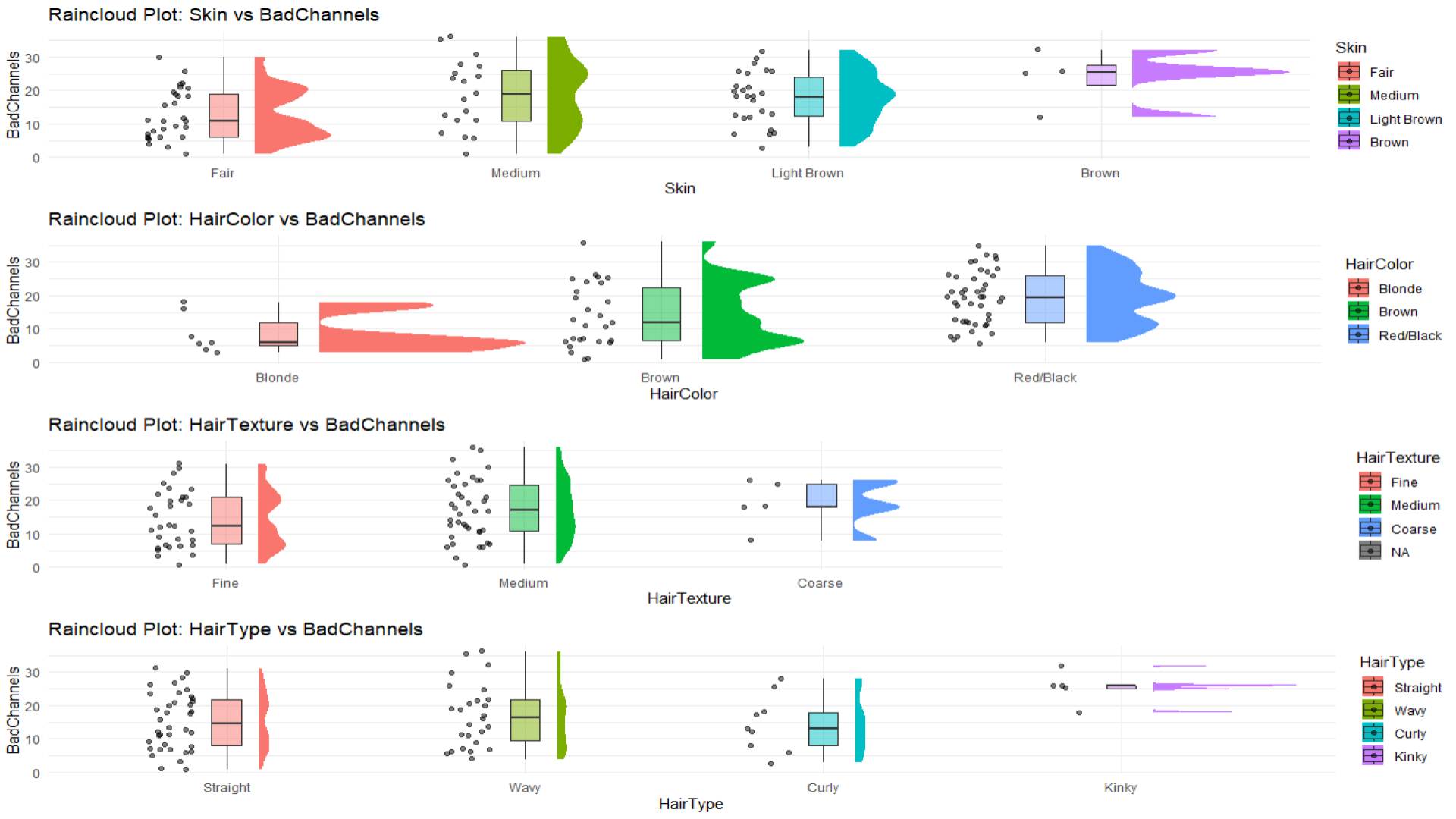


**Supplementary Figure 15**. Raincloud plots of the number of bad channels as a function of skin color

and hair color, texture, and type.

.
